# Metacognitions or distress intolerance: The mediating role in the relationship between emotional dysregulation and problematic internet use

**DOI:** 10.1016/j.abrep.2017.10.004

**Published:** 2017-10-26

**Authors:** Mehdi Akbari

**Affiliations:** Department of Clinical Psychology, Faculty of Psychology and Education, Kharazmi University, Tehran, Iran

**Keywords:** Metacognitions, Distress intolerance, Emotional dysregulation, Problematic internet use

## Abstract

**Objective:**

Given the relevance of problematic Internet use (PIU) to everyday life, its relationship to emotional dysregulation and the importance of metacognitions and distress intolerance in process and intermediaries research, this study examined which of metacognitions and distress intolerance acts as an intermediary between emotional dysregulation and PIU.

**Methods:**

In the current study, 413 undergraduate students from the University of Tehran, Iran (202 females; mean age = 20.13) voluntarily completed a questionnaire package which included the Internet Addiction Test (IAT), Difficulties in Emotion Regulation Scale (DERS), Metacognitions Questionnaire 30 (MCQ-30(, and Distress Tolerance Scale (DTS). The data were then analyzed using structural equation modeling by LISREL software.

**Results:**

Significant correlations were found between PIU and emotional dysregulation and both distress intolerance and metacognitions (*P* < 0.001). Structural equation modeling and path analysis results fit well to the data (χ^2^/df = 1.73; *p* < 0.001; RMSEA = 0.05; SRMR = 0.04; CFI = 0.97; NFI = 0.95). The results of the mediational model indicated that emotional dysregulation has an indirect impact via metacognition (β = 0.31; SE = 0.02) and distress tolerance (β = − 0.60; SE = 0.03) on PIU. The analysis also revealed a significant direct impact of emotional dysregulation on PIU, although this impact is much less than the indirect impact. The variables in this model accounted for 62% of the variance in participants' PIU levels.

**Conclusion:**

The results of this study provide evidence for the impact of emotional dysregulation on PIU through metacognitions and distress intolerance. Also, these findings emphasize that distress intolerance has a more significant mediating role than metacognition in the relationship between emotional dysregulation and PIU.

## Highlights

• This is the first study to explore the mediation role of distress intolerance in the relationship between emotional dysregulation and Problematic Internet Use (PIU).
• Relationships between distress intolerance and PIU were supported.
• The finding of this study indicate that distress intolerance plays a more significant mediating role than metacognition in the relationship between emotional dysregulation and PIU.
• Targeting distress intolerance may help reduce PIU.

## 1 Introduction

As a readily accessible source of information and entertainment for people of all ages, the Internet has become one of the primary necessities of life in almost all countries. Despite the various advantages bestowed by the World Wide Web, misuse of this technology can be dangerous and lead to Internet addiction (Li & Chung, 2006).

Problematic Internet Use (PIU), or excessive Internet use, is characterized by excessive or poorly controlled preoccupations, urges, or behaviors regarding computer use and Internet access that lead to impairment or distress (Young, 1996). PIU has been extensively researched since the mid-1990s, particularly in the Western and Asian countries. Although considerable evidence shows that PIU is associated with a number of negative health outcomes in adolescents and adults (Ko et al., 2012, Kuss et al., 2013), it was not officially classified as a clinical disorder in the latest edition of the Diagnostic and Statistical Manual of Mental Disorders (DSM-5) (American Psychiatric Association, 2013). This omission indicates the need for further evidence on this emerging mental health epidemic (Young, 2016).

Tokunaga and Rains (2010) used structural equation modeling to compare possible etiologies based on correlations derived from meta-analyses of a range of studies. They tested a “clinical” model in which psychosocial problems led to Internet use with Internet “problems” as the outcome as well as a non-clinical “self-regulation” model in which psychosocial problems predicted PIU, which in turn led to time spent using the Internet. According to Tokunaga and Rains, the finding that time spent on the Internet predicted PIU and not the other way around supports the non-clinical self-regulation model as well as the view that generalized PIU is not a clinical issue, but instead, a result of poor self-regulation. In other words, general problematic Internet behaviors appear to be less about the Internet itself and more about poor self-regulation.

A possible explanation as to how Internet use may become problematic lies in it taking the form of a maladaptive self-regulatory strategy (LaRose et al., 2003, Spada et al., 2008) rather than merely being used for problem-solving, entertainment and challenge (Caplan, 2007, Wan and Chiou, 2006). In support of this view, several studies have shown relationships between individual differences in automatic and controlled aspects of self-regulation and PIU (Billieux & Van der Linden, 2012). In recent years, increasing focus has been given to emotional dysregulation as a potentially transdiagnostic process of many forms of psychopathology. Emotional regulation has been defined as processes that serve to intensify, dampen, or maintain the behavioral, cognitive, experiential, or physiological aspects of emotion depending on an individual's goals (Gross & Thompson, 2007). Subsequent research has supported this conceptualization by demonstrating the role of emotional dysregulation in a wide range of clinical disorders (e.g., Lynch et al., 2007, Nolen-Hoeksema et al., 2008, Williams et al., 2012) and PIU (Caplan, 2010, Casale et al., 2016, Yu et al., 2013). Some researchers have argued that individuals who have emotional dysregulation are more likely to engage in addictive behaviors in an attempt to avoid or minimize negative emotions and try to alleviate distressing feelings (Yu, Kim, & Hay, 2013). Similarly, Hormes, Kearns, and Timko (2014) have observed that problematic users of social networking sites, compared to non-problematic ones, are more likely to experience emotional dysregulation.

The presence of a positive association between emotional dysregulation and PIU is not sufficient to clarify the psychological mechanisms that often lead a person with deficits in regulating emotions to engage in PIU. Research on the beliefs about the usefulness of the Internet for alleviating negative feelings might help in clarifying whether or not those who engage in the problematic use of the Internet are motivated to do so because they perceive Internet use as a useful strategy for managing distress (Spada et al., 2008).

Recent conceptualizations of addictive behaviors have also emphasized the role of metacognition in the genesis and perpetuation of emotional dysregulation (Spada et al., 2009, Spada et al., 2013). A growing body of research has emphasized the role of metacognitions as mediators in the association between emotional dysregulation and addictive behaviors (e.g. Spada, Caselli, Nikčević, & Wells, 2015). According to the metacognitive model, metacognition refers to cognition applied to cognition and may be defined as any knowledge or cognitive processes involved in the appraisal, control, and monitoring of thinking. It is purported that since metacognition fulfills an executive function with regard to cognitive processing, it also plays a contributory role in emotional dysregulation (Wells, 2000). Metacognitions refer to beliefs about the meaning of internal events and ways of controlling them. It is thought that such beliefs are central to the initiation and perseveration of unhelpful coping strategies (i.e. extended thinking, thought suppression, threat monitoring, avoidance, and maladaptive behaviors) which, in turn, lead to emotional dysregulation (Spada et al., 2008, Spada et al., 2015). Also, Spada and Marino (2017) showed that metacognitions might lead to the activation of maladaptive coping strategies, such as rumination and worry, which in turn may increase the likelihood of utilizing the Internet as a means of cognitive-affective self-regulation. The results from this study provide an essential addition to the literature on PIU, suggesting that both the emotional regulation model and the metacognitive model might be used to develop a theory-driven conceptualization of PIU and associated treatment.

The emergence of emotion theories of psychological dysfunction (e.g., Gross, 1998) has led to a growing interest in the characteristics of emotion and its regulation. Recent work by Simons and Gahar (2005), applying Gross's (1998) emotional regulation paradigm, has highlighted the potential role of “distress intolerance” in the development and maintenance of psychological dysfunction (Ameral et al., 2014, Anestis et al., 2012, Leyro et al., 2010, McHugh et al., 2014, Simons and Gaher, 2005, Zvolensky et al., 2011, Zvolensky and Otto, 2007, Zvolensky et al., 2010). More recently, a particular line of theoretical work has begun emphasizing the role of distress intolerance as central to the development and persistence of psychological dysfunction (Ameral et al., 2014, McHugh et al., 2014, Zvolensky et al., 2010).

Distress tolerance reflects an individual's perceived or behavioral capacity to withstand experiential or subjective distress related to affective, cognitive, and physical states (e.g., negative affect, physical discomfort;Simons and Gaher, 2005, Zvolensky et al., 2011). Simons and Gaher (2005) suggested that affective distress tolerance is multidimensional in nature, involving an individual's anticipation of an experience with negative emotions, including (a) ability to tolerate; (b) assessment of the emotional situation as acceptable; (c) how the individual regulates her/his emotion; and (d) how much attention is absorbed by the negative emotion and how much it interferes with functioning.

Individuals with low levels of distress tolerance tend to experience negative affect as intense, disruptive, and unacceptable and tend to engage in behaviors aimed at reducing feelings of distress. It has been suggested that low levels of trait distress tolerance may potentially lead to adverse outcomes (e.g. substance abuse) as individuals attempt to use maladaptive behaviors to cope with negative affect (Simons and Gaher, 2005, Zvolensky et al., 2011).

Although previous research has shown that PIU is associated with adverse outcomes, most of these studies have not tried to shed light on the underlying mechanisms that lead to PIU. When people have difficulty with emotional regulation and delayed gratification, they often turn to the Internet for distraction. Thus, we propose that an increasing number of people, particularly university students, are less capable of withstanding a negative psychological state such as boredom and loneliness when they do not receive sufficient stimulation from the environment to maintain optimum arousal levels. The ability to deal with such a state is referred to as distress tolerance (Simons and Gaher, 2005, Zvolensky et al., 2011). When tolerance is low, we hypothesize that students will attempt to escape distressing situations by using mobile devices or a computer to browse the Internet. To this end, these individuals will use the Internet to control the amount of stimulation they receive, thereby providing relief from the distress. Furthermore, the habitual use of the Internet as an escape from distressing situations may lead to poor academic performance observed in those university students who experience PIU.

As is evident form the literature review, distress intolerance plays a significant role in the development and maintenance of some of the psychopathologies, but to date, no attempt has been made to investigate the possible links between PIU and distress intolerance. Moreover, because the mediation role of metacognitions in the relationship between emotional dysregulation and PIU has been supported by previous research, the current study seeks to assess for the first time the mediating role of distress intolerance in the relationship between emotional dysregulation and PIU and to compare it with the mediating role of metacognitions. Based on the above, the hypotheses of the current research are as follows:

(1) Metacognitions have direct and indirect impacts (through emotional dysregulation) on PIU.

(2) Distress intolerance has direct and indirect impacts (through emotional dysregulation) on PIU.

## 2 Methods

### 2.1 Participants

The current study was a cross-sectional study examining the direct and indirect effects (intermediate effects) of a set of variables. A sample size of 437 students was calculated using Krejcie and Morgan's table with a 95% trust rate and 10% loss rate. Inventories were distributed to 437 undergraduate students from the University of Tehran, Iran. The students were selected by a convenience sample in the spring of 2017. General information about the purposes of the research project was announced to participants. The selected students completed the questionnaire package, which included the Internet Addiction Test (IAT), Difficulties in Emotion Regulation Scale (DERS), Metacognitions Questionnaire 30 (MCQ-30(, and Distress Tolerance Scale (DTS). After eliminating incomplete inventories, 413 inventories (202 females; mean age = 20.13) entered the final analysis.

### 2.2 Measures

#### 2.2.1 The Internet Addiction Test (IAT)

The Internet Addiction Test (IAT; Young, 1998b) consists of 20 items assessing the degree of PIU. This questionnaire includes 20 questions with a five-point Likert response format (i.e., rarely, sometimes, often, very often, and always). Scoring for the IAT is as follows: scores range from 20 to 100 with higher scores representing higher levels of PIU. Scores ranging from 20 to 39 indicate a medium rate of Internet addiction, 40 to 69 a high rate, and 70 to 100 a severe rate. This scale has been found to possess good psychometric properties (Widyanto & McMurran, 2004).

#### 2.2.2 Difficulties in emotion regulation scale (DERS)

This scale was designed by Gratz and Roemer in 2004 to measure emotional disorder and emotional self-regulation strategies and has 36 items on a five-degree Likert scale. The Cronbach's alpha coefficient was reported as 0.93, and the biweekly retested reliability coefficient was reported as 0.85 (Gratz & Roemer, 2004). The reliability of the Persian version developed by Asgari et al. through internal consistency was reported to be 0.86, and concurrent validity of the inventory was confirmed by the Beck Depression Scale and Multidimensional Pain Inventory (MPI) (Asgari, Pasha, & Aminian, 2009).

#### 2.2.3 Metacognitions questionnaire 30 (MCQ-30)

The MCQ-30 is a self-report measure that assesses individual differences in metacognitive beliefs, judgments and monitoring tendencies. It consists of five replicable subscales assessed by 30 items in total. The five sub-scales measure the following dimensions of metacognition: (1) positive beliefs about worry; (2) negative beliefs about worry concerning uncontrollability and danger; (3) cognitive confidence; (4) beliefs about the need to control thoughts; and (5) cognitive self-consciousness. Each item is rated on a four-point Likert scale, ranging from 1 (disagree) to 4 (strongly agree). Higher scores indicate higher levels of unhelpful metacognitions. The MCQ-30 possesses good psychometric properties (Spada et al., 2008).

#### 2.2.4 Distress tolerance scale (DTS)

The DTS measures distress tolerance (Simons & Gaher, 2005). This scale is a 15-item self-report measure that examines one's perceived ability to tolerate emotional distress and includes questions related to tolerance, appraisal, absorption, and regulation. Initial exploratory and confirmatory factor analyses of the DTS by Simons and colleagues (2005) among large college-aged samples (Study 1: 642 students; Study 2: 823 students) supported a four-factor model composed of four subscales: tolerance (α = 0.72) (“perceived ability to tolerate emotional distress”), appraisal (α = 0.82) (“subjective appraisal of distress”), absorption (α = 0.78) (“attention being absorbed by negative emotions”), and regulation (α = 0.70) (“regulation efforts to alleviate distress”) with good test-retest reliability (intra-class *r* = 0.63). The total DTS score was used for this study since it has better internal consistency than the four scales (Leyro et al., 2011; Simons & Gaher, 2005). The total DTS score has good convergent and discriminant validity in relation to negative affect, nicotine dependence, and smoking beliefs (Leyro et al., 2011).

### 2.3 Data analysis

Self-reported data on IAT, DTS, DERS, and MCQ-30 from a sample of 413 participants were analyzed using structural equation modeling. Structural Equation Modeling (SEM) was performed to test the hypothesized effects of DERS on IAT through metacognitions and distress intolerance. We used LISREL 8.80 software (Jöreskog & Sörbom, 2006) with maximum likelihood estimation. To assess the overall fit of both models, we used the χ^2^ to degrees of freedom (df) ratio, Comparative Fit Index (CFI), Normed Fit Index (NFI), Goodness of Fit Index (GFI), relative fit index (RFI), Standardized Root Mean Squared Residual (SRMR), and Root Mean Square Error of Approximation (RMSEA). A model can be considered to fit the data if χ^2^/df < 2; RMSEA < 0.05; SRMR < 0.08, CFI ≥ 0.90 to 0.95, IFI ≥ 0.90 to 0.95, NFI ≥ 0.90 to 0.95, and RFI ≥ 0.90 to 0.95. Indirect effects were tested with a distribution of product coefficients (P) test developed by MacKinnon, Lockwood, Hoffman, West, and Sheets (2002).

## 3 Results

Descriptive statistics (i.e., mean, standard deviation) and correlations among variables are presented in Table 1. In this study, 5.5% of the inventories with incomplete responses were put aside. The assumption of normality of all four scales was confirmed using the Kolmogorov-Smirnov test. The correlations between PIU and emotional dysregulation, distress tolerance, and metacognitions were significant (*P* < 0.001) (Table 1). The Internet Addiction Test results indicate that 57% of the students were normal users, 33% of them were exposed to PIU, and 10% of them were classified as being addicted to the Internet.

**Table 1:** Means, standard deviations, and correlation matrix of the research variables.

Variables	M (SD)	1	2	3	4
1. IAT- PIU	46.41 (3.04)	1			
2. Emotional dysregulation	93.11 (7.64)	0.49⁎⁎	1		
3. Metacognitions	64.23 (5.11)	0.57⁎⁎	0.43⁎⁎	1	
4. Distress tolerance	35.95 (3.27)	− 0.68⁎⁎	− 0.55⁎⁎	− 0.19⁎	1

Notes: *N* = 413.

⁎ *p* < 0.05.

⁎⁎ *p* < 0.001.

### 3.1 Structural equation modeling

Beta coefficients and significance status of direct and indirect impacts of variables are shown in Table 2. All relationships between variables were significant.

**Table 2:** Structural equation model.

	β	SE	T	P
Direct impact				
Emotional dysregulation on PIU	0.12	0.06	2.49	0.01
Emotional dysregulation on metacognitions	0.37	0.08	5.11	0.001
Emotional dysregulation on distress tolerance	− 0.56	0.07	− 8.64	0.001
Metacognitions on PIU	0.43	0.05	6.01	0.001
Distress tolerance on PIU	− 0.71	0.06	− 11.41	0.001
Indirect impact				
Emotional dysregulation via metacognitions	0.31	0.02		
Emotional dysregulation via distress tolerance	− 0.60	0.03		

### 3.2 Direct impact analysis

According to the results of the structural equation modeling, metacognitions have a direct impact (β = 0.43; *P* < 0.001) on PIU. Moreover, the direct impact of distress tolerance on PIU was significant (β = − 0.71; *P* < 0.001). The results of the SEM indicate that emotional dysregulation has a direct impact on PIU (β = 0.12; *P* < 0.01).

### 3.3 Mediation analysis

**Fig. 1:**
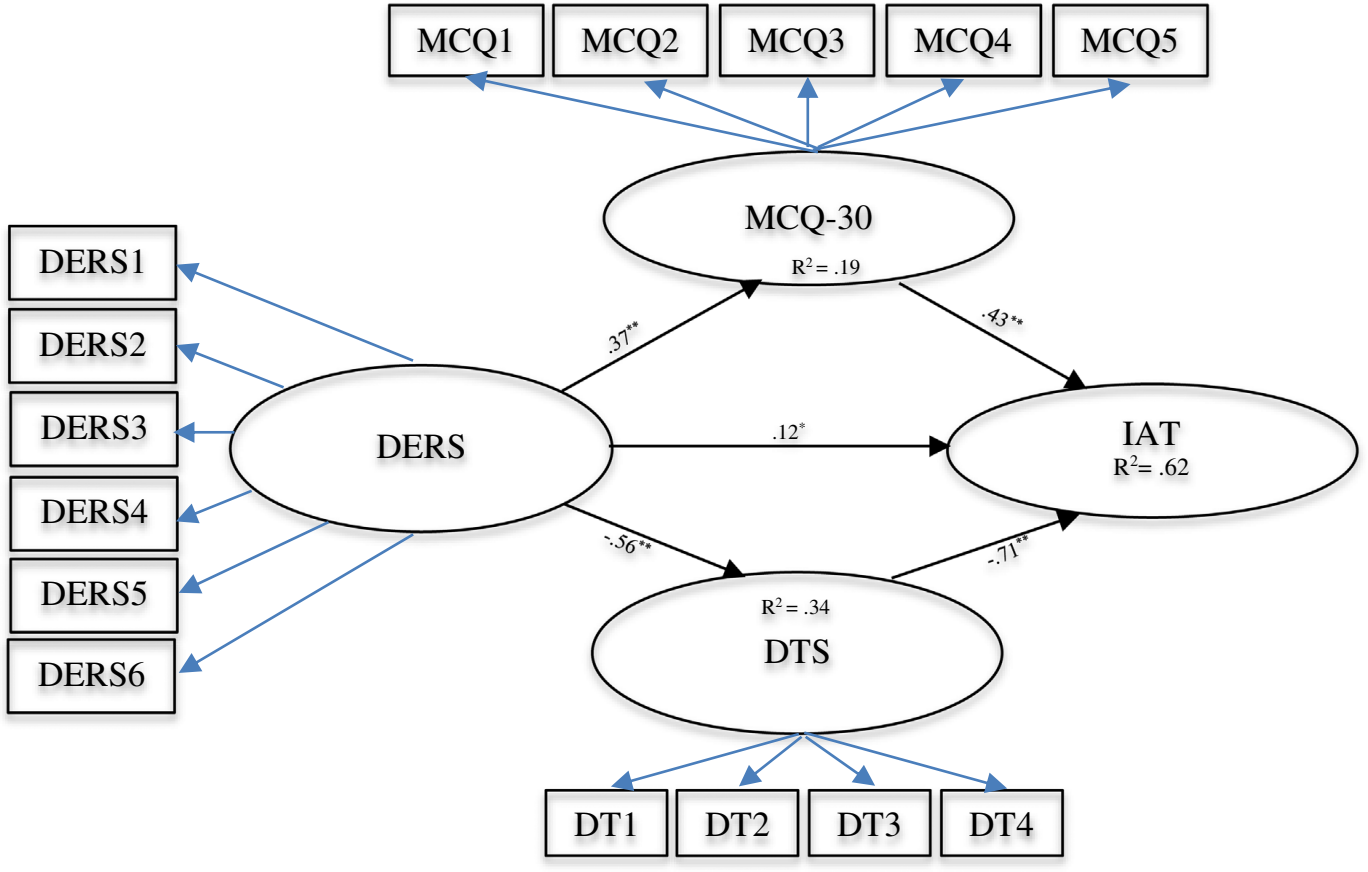
Results of structural equation modeling analysis of the direct and indirect impacts of emotional dysregulation and the mediating role of metacognitions and distress tolerance on PIU.

The results of the mediational model presented in Table 2 indicated that emotional dysregulation has an indirect impact via metacognitions (β = 0.31; SE = 0.02) and distress tolerance (β = − 0.60; SE = 0.03) on the PIU. According to the results of the SEM, as hypothesized metacognitions and distress intolerance mediate the role of the emotional dysregulation on PIU. A simplified diagram of the non-mediational and mediational models is depicted in Fig. 1. Factor loadings of all latent variables are not depicted for simplicity's sake. The beta coefficients and the relationships between variables are also presented in Fig. 1. The results support the hypothesized indirect relationship between emotional dysregulation and PIU levels mediated by both metacognitions (*P* = 24.12; *p* < 0.05) and distress tolerance (*P* = − 41.80; p < 0.05). The analysis also reveals a significant direct impact of emotional dysregulation on PIU. The variables in the model accounted for 62% of the variance in participants' PIU levels.

### 3.4 Measurement model

The analysis of the structural model of PIU produced good indices of fitness. The fitness indices for the model are reported in Table 3. As depicted in Table 1, the chi-square index was statistically significant, and incremental indices (CFI) obtained values of 0.95. or higher. RMSEA, its *p*-value for close fit, and SRMR indicate a good overall fit. All parameters were statistically significant.

**Table 3:** Model fitness examination indices.

χ^2^/df	CFI	NFI	GFI	IFI	RFI	SRMR	RMSEA
1.73	0.97	0.95	0.96	0.95	0.95	0.04	0.05

*Note. MCQ1: positive beliefs; MCQ2: uncontrollability and danger; MCQ3: cognitive confidence; MCQ4: Need to control thoughts; MCQ5: cognitive self-consciousness; DERS1: non-acceptance of negative emotional responses; DERS2: difficulties engaging in goal-directed behavior when distressed; DERS3: difficulties controlling impulsive behaviors when distressed; DERS4: lack of emotional awareness; DERS5: limited access to effective ER strategies; DERS6: lack of emotional clarity; DT1: tolerance; DT2: appraisal; DT3: absorption; DT4: regulation; IAT: Internet Addiction Test;* ^⁎⁎^*p* < *0.001;* ^⁎^*p* < *0.01*.

## 4 Discussion

The goal of this research is to study the direct and indirect effects of metacognitions and distress intolerance on PIU and to compare the mediating role of these variables in PIU. Analysis of the research data shows that metacognitions have a mediating role in relation to emotional dysregulation and PIU. The results indicate that emotional dysregulation has an indirect impact via metacognitions (β = 0.31; SE = 0.02; *P* = 24.12, *p* < 0.05) on PIU. Moreover, the results show that the mediator path through metacognition determined 19% of PIU variance.

This finding is consistent with those of previous studies, such as Spada et al. (2008), Hormes, Kearns, and Timko (2014), Yu et al. (2013), and Casale et al. (2016) that demonstrated the effect of metacognitions and emotional dysregulation on PIU. The findings confirm that the presence of metacognitions may lead to the activation of maladaptive coping strategies, such as rumination and worry, which in turn may increase the likelihood of utilizing the Internet as a means of cognitive-affective self-regulation. In other words, using the Internet may become a strategy to control unwanted negative emotions. Although the results of the current study partially confirm the findings of the studies mentioned above, what sets this study apart is the evidence for metacognitions playing a partial mediating role between emotional dysregulation and PIU.

This research also aimed to examine the mediating role of distress intolerance in the relationship between emotional dysregulation and PIU. The results of this study support the mediating role of distress tolerance between emotional dysregulation and PIU. The results indicate that emotional dysregulation has an indirect impact via distress tolerance (β = − 0.60; SE = 0.03; *P* = − 41.80, *p* < 0.05) on PIU. The results also suggest that the mediating role of distress intolerance is more significant than that of metacognitions. Furthermore, the results show that the mediator path through distress tolerance determined 34% of PIU variance.

This study is the first to examine the mediating role of distress intolerance in the relationship between emotional dysregulation and PIU. Interest in distress intolerance in the context of psychological disorders has been paralleled by the growth and dissemination of psychosocial interventions designed to promote tolerance for distress originating from internal and external sources (e.g., physical discomfort, stressful life events) (Bardeen et al., 2013, Ellis et al., 2013). Thus, as a global factor, distress tolerance may be thought of as being related to the influence of the evaluation and consequences of exposure to aversive stimuli and related adaptive and maladaptive behavioral responses such as PIU. This perspective on distress tolerance and related processes reflects the theoretical context for the empirical study of the potential relations between distress tolerance and risk and resilience to various forms of psychopathology (Zvolensky et al., 2011).

As previously mentioned, distress tolerance reflects an individual's perceived or behavioral capacity to withstand experiential/subjective distress related to affective, cognitive, and/or physical states (e.g., negative affect, physical discomfort) (Simons and Gaher, 2005, Zvolensky et al., 2011). Although distress intolerance is presumably related to other emotional vulnerability processes such as anxiety sensitivity, emotional dysregulation, withdrawal phobia, and experiential avoidance among others, the available work suggests it is a unique psychological construct. For example, based on extant biopsychosocial models and empirical evidence focused on distress intolerance, people with elevated levels of perceived intolerance for negative emotional events may tend to be more emotionally reactive to stressors when coping with emotionally distressing events, thoughts, and may try to escape or avoid them (e.g. coping-oriented motives for cannabis use; Leyro et al., 2010).

When tolerance is low for situations that produce a negative psychological state, it is hypothesized that people will attempt to escape distressing situations by using mobile devices or a computer to browse the Internet. To this end, individuals may use technology to control the amount of stimulation they receive, thereby providing relief from the distress.

The presented results are preliminary, and some limitations should be highlighted. First, the sample was not randomly selected, and the use of data from a self-report measure is typically influenced, to some degree, by recall bias and answer accuracy. Second, the cross-sectional design employed does not allow definitive statements about causality. Longitudinal studies are needed to clarify the direction of the associations highlighted by the current research. Indeed, the cross-sectional design does not allow conclusions to be drawn about the direction of the association, and it is not possible to rule out that emotional dysregulation is at least, in part, a result of the excess use of the Internet at the expense of real situational modeling of appropriate affect regulation. Another limitation of the current study is that PIU was not investigated in the context of specific Internet activities. It is likely that the types of cognitive psychopathologies vary among the wide range of Internet users groups. Therefore, it is suggested to study this variation in the context of Internet-based specific activities to determine the types of cognitive psychopathology associated with each group in the future studies.

In conclusion, the results of this study showed that distress intolerance plays a full mediating role and metacognitions play a partial mediating role between emotional dysregulation and PIU. The results of this study have potentially significant implications for developing prevention and intervention programs for adolescents with PIU. Therefore, it may be useful to develop interventions that take into account how both distress intolerance and emotional dysregulation may lead to PIU. These findings provide the first step in expanding the distress intolerance literature on PIU. Although this study found a significant association between PIU and low distress tolerance, this work should be replicated. If these findings hold, they could have significant treatment implications for developing a theory-driven conceptualization of PIU and associated treatment based on both the emotional dysregulation model and the distress intolerance model.
